# The Clash of Microbiomes: From the Food Matrix to the Host Gut

**DOI:** 10.3390/microorganisms10010116

**Published:** 2022-01-06

**Authors:** Despoina Eugenia Kiousi, Nikos Chorianopoulos, Chrysoula C. Tassou, Alex Galanis

**Affiliations:** 1 Department of Molecular Biology and Genetics, Democritus University of Thrace, 68100 Alexandroupolis, Greece; dkiousi@mbg.duth.gr; 2 Institute of Technology of Agricultural Products, Hellenic Agricultural Organization—DIMITRA, Lycovrissi, 14123 Attiki, Greece; nchorian@nagref.gr (N.C.); ctassou@nagref.gr (C.C.T.)

**Keywords:** functional foods, microbiome, probiotics, fermentation, lactobacilli, multi-omics

## Abstract

Food fermentation has led to the improvement of the safety characteristics of raw materials and the production of new foodstuffs with elevated organoleptic characteristics. The empirical observation that these products could have a potential health benefit has garnered the attention of the scientific community. Therefore, several studies have been conducted in animal and human hosts to decipher which of these products may have a beneficial outcome against specific ailments. However, despite the accumulating literature, a relatively small number of products have been authorized as ‘functional foods’ by regulatory bodies. Data inconsistency and lack of in-depth preclinical characterization of functional products could heavily contribute to this issue. Today, the increased availability of omics platforms and bioinformatic algorithms for comprehensive data analysis can aid in the systematic characterization of microbe–microbe, microbe–matrix, and microbe–host interactions, providing useful insights about the maximization of their beneficial effects. The incorporation of these platforms in food science remains a challenge; however, coordinated efforts and interdisciplinary collaboration could push the field toward the dawn of a new era.

## 1. The Advent of Fermented Foods and Their Functional Applications

The concept of fermented foods originates from ancient years, as our ancestors tried to stretch out the life expectancy of raw materials and improve their safety characteristics. Indeed, fermented foods and beverages are more resistant to spoilage, as they are a hostile environment for pathogen expansion and can maintain their quality characteristics at ambient temperatures. Thus, fermentation can be considered a form of bio-preservation. Notably, the advent of fermentation techniques happened pan-continentally. Meat, wheat, and dairy fermented products using lactic acid bacteria (LAB), molds, and yeasts were mainly performed in Europe, Australia, and North and South America, while people on the African continent mainly used root vegetables and milk. In Asia, ancient populations were utilizing vegetables, soybean, rice, and fish as substrates for fungi and bacilli to produce alcoholic beverages, such as sake, and fish sauce [1]. Today, modern science has helped us overcome the problem of raw material preservation; however, fermented foods are still being faithfully consumed. The amplified appeal of fermented foods can be attributed to two factors. Firstly, raw material fermentation can produce new textures and flavors contributing to the hedonistic value of food, and secondly, the intake of these foodstuffs has been intuitively linked to the well-being of the consumers [2].

Fermented foods that are thought to provide a health benefit to the consumer are often referred to as “functional foods” or “nutraceuticals” [3]. These foods can either be fortified with vitamins and bioactive compounds, such as phytochemicals, or contain probiotic microorganisms [4]. Consumer interest in these products has risen steeply in recent years, as the concepts of wellness and disease prevention are gaining more attention in Westernized countries. However, studies on the effect of functional foods on animal and human health are limited, oftentimes confusing, and even contradictory [5]. Therefore, regulatory frameworks have imposed strict guidelines about which products can be labeled as such. In 1991, Japan was the first country to implement legislative frameworks to control the growing market needs and protect consumers from fraud [6]; subsequently, the commercialization of functional foods in Japan requires the “Food for Specified Health Uses” (FOSHU) approval, awarded by the Japanese Ministry of Health, Labour and Welfare. In 2020, 1071 products received FOSHU permission [7]. In Europe, on the other hand, a significantly smaller number of functional foods have been authorized by the European Food Safety Authority (EFSA) [8].

From this perspective, we will critically discuss the current situation regarding fermented functional food products and provide new insights for the comprehensive characterization of their potential health benefits to consumers. Recent advances in omics and their increased accessibility have offered the opportunity to systematically characterize the composition and functional aspects of fermented products, as well as their effect on consumers. However, their incorporation into food science studies remains a challenge.

## 2. Breaking Down the Fermentation Process

Fermentation is defined as the process in which a substance breaks down into a simpler substance. More specifically, fermentation is a form of anaerobic metabolism, taking place in the absence of molecular oxygen under favorable temperature and pH conditions [9]. The substrates of these reactions are complex carbohydrates present in meat, cereal, legumes, fish, vegetables, and fruits that can be broken down to produce alcohols, carbon dioxide, ammonia, fatty acids, and other compounds [2]. The catalyzer of these reactions is usually autochthonous or allochthonous bacteria and fungi that can use raw materials as energy sources. Indeed, LAB, including *Lactobacillus* (and the newly excised genera *Lacticaseibacillus*, *Lactiplantibacillus*, *Lentilactobacillus*, *Lavilactobacillus*, and *Limosilactobacillus*), *Leuconostoc*, and *Streptococcus*, *Saccharomyces cerevisiae*, and molds are being predominantly applied to produce the vast array of available dairy and non-dairy products. These microorganisms are usually organized in consortia, termed starter cultures, that are explicitly introduced in specific raw materials. For example, yoghurt-associated microbes are usually strains of the *Lactococcus*, *Leuconostoc*, *Pediococcus*, and *Lactobacillus* genera, whereas meat-fermenting bacteria typically belong to the *Lactobacillus* and *Weissella* genera or are coagulase-negative staphylococci [1].

Fermentation byproducts include several bioactive compounds that elevate the nutritional and functional profile of the foodstuffs. For example, lipoteichoic acid produced by *Bifidobacterium animalis* subsp. *lactis* BPL1 was shown to reduce obesity-related biomarkers in preclinical models [10]. Other compounds of interest include active forms of phenolic compounds, as well as antimicrobial substances, such as bacteriocins [11]. LAB can also produce exopolysaccharides, short chain fatty acids (SCFAs), and vitamins [12]. It is important to note, however, that these compounds are usually present in small quantities, and thus it is difficult to predict whether healthy populations will benefit from them. Furthermore, another important aspect of fermentation is that bacteria can metabolize and inactivate toxic compounds, such as phytic acid, a metal-chelating compound that acts as an anti-nutrient [13]. Interestingly, these compounds may be produced by chain reactions, in which more than one bacterial or fungal species participate, and thus the composition of the microbial community is of integral importance.

## 3. Fermented Foods Harbor a Stable, Yet Dynamic, Microbial Ecosystem

Fermented products present a rich microbial ecology, being comprised of stable communities. The impact of this microbial fingerprint on the organoleptic and functional properties of the products is pivotal, and thus the stability of its composition must be ensured throughout production and storage [14]. Furthermore, the stability of populations is a safety prerequisite imposed on novel functional products by regulatory bodies [15]. Changes in these dynamics or the establishment of invading species could result in altered organoleptic characteristics and even spoilage [16]. Indeed, unstable ecological dynamics may lead to the expansion of prolific lineages at the expense of other populations [17]. The homeostatic composition of these food matrices is termed the food microbiome. The structure of these communities can be studied using conventional culture-based methods; however, the advent of next generation sequencing platforms has simplified this process and has offered the opportunity to study uncultivable strains during production and storage. In this context, it has been demonstrated recently that next-generation sequencing platforms may be more efficient in identifying spoilage microbiota than conventional techniques [18].

Some residents of the food microbiome are characterized as probiotics, live microorganisms that can reach the intestine and confer a health benefit to the host when administered in a sufficient amount. The definition was proposed by FAO/WHO [14] and was later updated by Hill et al. [15]. Intake of probiotics may result in the modulation of the gut microbiome [19] or the immune system of the host [20], while several reports also indicate potential alleviatory effects against certain gastrointestinal conditions [21], enhancement of nutrient bioavailability, and prevention of allergy onset in susceptible individuals [22]. Generally, probiotics belong to the *Bifidobacterium* or the basonym *Lactobacillus* genera and are mainly isolated from human or animal internal cavities [23] and dairy or non-dairy food products [24,25]. Probiotics have been used in fermentation for centuries, thus becoming domesticated in specific food matrices. On this note, lactobacilli can be adapted to a broad range of environmental niches due to their heightened genetic diversity. Lactobacilli species can be categorized based on their fermentation characteristics as facultative or obligate heterofermentative or as obligate homofermentative [26]. This distinction is based on their preferred pathways for carbohydrate, mainly hexose, fermentation and their ability to utilize amino acids from the environment. However, a question that arises is what happens when we try to introduce a new strain of interest in established food microbiomes. This topic is exceptionally relevant for the functional food industry.

Evolving artificial communities to welcome new strains is an inherently complex matter that is governed by population dynamics. Several studies have tried to simulate microbe–microbe interactions [17] and trophic relations [27] that occur in the food matrix. Indeed, community selection experiments have shown that during a process called neutral drift, microorganisms may gain or lose traits that can help them adapt and populate a specific matrix. In case the new traits are taxing, mutants can be established, leading to changes in the microbial fingerprint [28]. Antagonistic activity between strains can also be observed in terms of nutrient availability or the production of antimicrobial compounds, such as bacteriocins [29]. Lactobacilli are avid producers of a wide array of bacteriocins that can target gram-positive and/or gram-negative strains, rendering them ineffective. The producer strains are generally immune to these proteins, and thus they do not suffer the negative effects of their production [30]. Subsequently, the resident microbiota in the food matrix or the host can induce these effects in strains of interest and thus limit their viability and functionality. In the best-case scenario, LAB can be smoothly incorporated in the food matrix and develop mutualistic relationships with other residents of the food microbiome. In this context, lactobacilli may opt for pentose and not hexose metabolism in the presence of yeasts, which preferentially metabolize pentoses. Therefore, niche partitioning takes place, benefiting both lactobacilli and yeasts, two microorganisms commonly found in starter cultures [26]. Bacteria–fungi interactions are an integral part of a microbial ecosystem and are characterized by heightened molecular complexity [31]. The enhanced availability and accessibility of omics platforms supports the prediction of the behavior of probiotics in food matrices and their interactions with resident microbiota. However, only a handful of studies have incorporated these tools in the investigation of food microbiomes [16]. These more sophisticated approaches can save researchers a lot of time and resources and filter out strains that cannot adapt to these niche-specific conditions.

Lastly, population dynamics are strongly influenced by chemical and physical factors, such as the availability of nutrients, salinity, pH, and post-fermentation media acidification [32]. Furthermore, the high pressure and extreme temperature prevailing in the food industry can also alter the composition of microbial consortia [33]. The survival and function of probiotic strains can also be compromised by these factors, and thus it is of integral importance to ensure their stability in functional products. In this context, regulatory bodies require a minimum viable concentration of 10^6^ CFU/mL of probiotics in functional products [14]. Viable count estimation can be performed by conventional culture-based techniques. Notably, a recent study proposed a novel method for viable count enumeration using Fourier-transform infrared spectroscopy and machine learning [34]. The enhancement of viability of probiotic strains in foods is a topic that has garnered a lot of attention, and thus strategies, such as the incorporation of prebiotic fibers or encapsulation methods, have been established [32].

## 4. The Clash of Microbiomes

When ingesting fermented products, the microbiome of the food matrix meets the ‘known unknown’ of the host microbiome. The interactions that occur thereafter are of great interest; however, we may not be able to fully understand their extent [35]. The problem is that the gut microbiome cannot be fully recapitulated in vitro due to its high complexity and host specificity. Additionally, we have yet to characterize a specific microbiome composition as ‘homeostatic’ or ‘healthy’. Therefore, it is hard to model something we have little knowledge about. Furthermore, the host’s genetics, lifestyle, ethnicity, age, and pathophysiology can have a tremendous effect on microbiome composition and function [36], adding more layers of complexity. However, some simplistic models to study the evolutionary relationships and dynamics between microbiome residents have already been established. Baroso-Batista et al. established a mouse model to study *Escherichia coli* evolution after mice mono- or co-colonization with other bacteria. It was shown that the evolutionary patterns of *E. coli* differed in each case, and that specific genes and metabolic pathways were selected for its adaptation [37]. These studies might help us understand and predict the phenotypical changes that an ingested strain may go through during the transition from the microbial communities of food to those of the gut. In the same context, host factors can also determine ecological interactions. More specifically, it was shown that young mice exerted different evolutionary pressures to the strains than their aged counterparts [38]. Concerning the genetic fingerprint of the host, specific polymorphisms in genes coding for proteins involved in pathogen responses and immunological function can exert evolutionary pressures on the gut microbiota, influencing heritable lineages and microbial abundance [39]. These studies confirm that the incorporation of extrinsic strains in a microbiome niche is dependent on several environmental factors influencing the metabolic features of newcomer strains and therefore altering their functionality in this new environment. Importantly, computational methodologies have been developed for the spatiotemporal study of these interactions, aiding in the understanding of the intricate interactions taking place in complex microbial communities and providing fertile ground for interdisciplinary collaboration [40].

Probiotic strains are already incorporated into the fabric of the human microbiome in several niches, as they can be found in the skin and the gastrointestinal and genitourinary tracts. These strains are adapted to thrive in these niches, something that is reflected in their genetic composition. One of the most characteristic examples of strictly host-adapted bacteria is *Lactobacillus iners*, which inhabits the female genitourinary tract. The genome of this bacterium is one of the smallest genomes of the basonym *Lactobacillus* genus, with a total size of 1.28 Mb, which is the result of extensive gene loss [41]. When a strain adapts to a nutrient-dense environment, such as that of the human niche, it experiences loss of amino acid biosynthesis clusters, as amino acids are readily provided by the host. Furthermore, bacteria are becoming biased toward consuming only a small array of carbohydrates, those abundant in their niche [26]. Strains of this species are not expected to withstand the conditions prevalent in the food industry due to their highly fastidious character [42]. On the other hand, lactobacilli that inhabit raw materials and fermented products, such as *Lactiplantibacillus plantarum*, *Lactiplantibacillus pentosus*, *Lacticaseibacillus casei*, and *Lacticaseibacillus rhamnosus*, generally present a wider array of biosynthetic capabilities, and these species are often termed as ‘free living’ or ‘nomadic’ [26]. Due to the higher genetic complexity of strains inhabiting fermented foods than that of their host-associated counterparts, it is expected that they may be able to, at least transiently, colonize the host. Indeed, experiments have shown that ingested probiotics can survive, transiently attach to the gut lining, and alter the host’s gene expression in a timely manner [43]. However, most strains are excluded by the gut microbiome after the cessation of treatments.

The reciprocal effect of ingested microbes on the resident microbiota is a topic of debate. Several animal and clinical studies have shown mild changes in gut microbiome composition after ingestion of probiotics; however, more pronounced differences can be observed in the metabolic function of the gut microbial consortia [44]. Concerning structural composition, two recent elegant studies showed that probiotics may delay the repopulation of the gut with resident microbiota after antibiotic treatments, probably due to the production of antimicrobial compounds by species of the basonym *Lactobacillus* genus [43,45]. This effect, however, may be negligible in homeostatic conditions in healthy adults. On the other hand, even mild changes in the structure of the microbiome can have an amplified effect on its metabolic function. One consequence that has been documented across several studies is that probiotics can enhance the populations of SCFA producers, leading to changes in the metabolic profile of the microbiome [46]. SCFAs have been linked with various physiological effects, such as immunomodulatory actions [47], and thus they can act as ‘messengers’ of changes in the gut microbiome that can systemically affect the host. Consequently, more studies are needed to investigate these complex interactions and to help predict the efficacy of probiotic supplementation.

## 5. Newcomers in Action

The consumption of fermented products has been empirically linked to beneficial effects on the health of the consumer [11]. The first systematic observation of these effects was performed by Elie Metchnikoff, who claimed that Caucasian villagers who consume large quantities of fermented milk products lead a healthier and longer life than their socioeconomical conditions would allow [48]. The vast majority of available clinical data on the effects of functional food consumption refer to positive outcomes in gastrointestinal health, and more precisely, against constipation and rectal bleeding [49]. These effects can be mediated by changes in microbiome composition and function, by the lowering of intestinal inflammation, or by the overall enhancement of bowel movements [50]. One of the most studied strains that has also been used at the industrial scale is *Lacticaseibacillus paracasei* strain Shirota (LcS), previously known as *Lactobacillus casei* strain Shirota, which was originally isolated from fecal samples. Recent studies have evaluated the effect of LcS-fermented dairy beverages against constipation [51], constipation-related symptoms in women after childbirth [52], and intestinal health in general [53]. These studies agree that the consumption of this fermented product may improve some outcomes in people with constipation. However, minimal changes in the protocol and measurement of outcomes do not allow for comparison of the studies and the generation of a solid conclusion. Furthermore, other studies have described the effect of LcS-fermented dairy products on depression outcomes in constipated adults. It is interesting to note that some of these studies did examine the effect of supplementation on gut microbiota composition. For instance, Zhang et al. found that LcS-fermented milk modified the gut microbiome structure, increasing the abundance of specific beneficial species while decreasing the abundance of species related to mental illness [54]. Although a positive correlation was observed, further studies are needed to elucidate the mechanism by which these effects were mediated and to establish a causal link between the supplementation and observed outcomes.

Another common health claim of functional foods is the lowering of blood cholesterol and/or blood pressure. More specifically, exopolysaccharides produced by bacteria present in the products can bind cholesterol and stimulate the production of bile acids, ultimately leading to decreased cholesterol absorption [11]. In this context, in a randomized triple-blind control study, patients with chronic heart failure were given low fat yoghurt containing *Lactobacillus acidophilus* La5 and *Bifidobacterium lactis* Bb12 or non-fortified yoghurt. The intervention resulted in a significant reduction of serum oxidized low-density lipoprotein (Ox-LDL) in the intervention group; however, no significant changes in other markers of cardiac function and overload were recorded. The authors postulated that the consumption of the foodstuff led to the induction of antioxidant effects, rather than direct regulation of blood cholesterol [55]. The same consortium of probiotics was also incorporated into fermented milk and administered to patients with type 2 diabetes mellitus. It was shown that the intervention group presented improved glycemic control and lower cholesterol levels compared to the control, while both groups showed a decrease in pro-inflammatory cytokines [56]. Interestingly, a different microbial consortium that included strain *B. lactis* Bb12 seemed to have a more direct effect in the lowering of triglyceride and increase in high-density lipoprotein cholesterol (HDL-c) [57]. These studies highlight that the vehicle for probiotic supplementation, as well as differences in the microbial composition of the tested foods, could affect the clinical outcomes.

The gut-brain-microbiome axis has been in the spotlight for the last few years, as the mechanisms that govern this interplay have started to be unraveled [58]. Thus, several studies tackle the effect of functional food consumption in the gut microbiome and subsequently in central nervous system activity. In this context, individuals with mild cognitive impairment were recruited to consume *L. plantarum* C29-fermeted soybeans to investigate its effect on cognitive function [59]. The authors found that the intervention improved markers of neurocognitive function, while changes in fecal lactobacilli content were also reported [59]. Accordingly, milk beverages enriched with LcS limited stress manifestation in students during university exams. These effects were attributed to the possible manipulation of the hypothalamus–pituitary–adrenal (HPA) axis [60]. Interestingly, lactobacilli can produce neurotransmitters that can reach the central nervous system to exert their effects [61]. For example, administration of milk fermented by *Lactobacillus brevis* DL1-11 led to the reduction of anxiety symptoms and insomnia in mice. This effect was attributed to the high amounts of the inhibitory neurotransmitter γ-aminobutyric acid that was secreted in the food matrix by the probiotic strain [62]. However, the metabolic profile of functional products and their content in such bioactive compounds should be further studied.

A common feature of the presented studies is that the composition of the starter cultures is not disclosed, while multiple strains are being tested at the same time. Subsequently, it is hard to decipher which strain has a potential impact on consumers’ health. Additionally, the interplay of these strains is usually not investigated. Furthermore, the chemical composition of the functional products in terms of bioactive compound content is rarely explored, and thus in case of positive outcomes, the mechanism is not adequately studied. Therefore, the use of lactobacilli to produce bioactive metabolic byproducts in the food matrix could be more complex than previously thought. The integration of omic technologies in food science can help researchers tackle these issues and enhance the efficacy of functional food supplementation in the clinical setting.

## 6. The Dawn of a New Era

The systematic characterization of the gut microbiome during the last few decades and the comprehensive investigation of its structure and function, as well as its effects on the host, had a positive effect on the study of microbial consortia, regardless of their origin. The study of the human microbiome was enabled by the invention of next generation sequencing platforms and the establishment of efficient computational pipelines to analyze the generated data. Today, the number of available fermented products is increasing exponentially due to heightened consumer interest. However, clinical claims in functional foods are rarely being made, even though these products have been empirically used for their proposed health-promoting properties. Data disparity and lack of translatability of preclinical findings to the clinic are vital contributors to this phenomenon. Indeed, preclinical studies that use cellular models do not take into consideration the biological complexity of the organisms, and thus findings about the effect of specific probiotics or their bioactive metabolites should be cautiously interpreted and validated in animal models and in human volunteers. For example, in vitro experiments of intestinal colonization fall short in the fact that they do not take into consideration the interplay of the probiotic strain with gut commensals and the immune system of the host. Importantly, the joint action of different strains is rarely being tested in vitro, and thus the mechanism of action of multispecies supplementation is not adequately described prior to the introduction to clinical studies. Concerning animal studies, structural differences of the murine gut microbiota and the lack of the level of heterogeneity presented in human hosts could also produce artifacts, underlying the need for more robust models [63,64]. Additionally, mechanistic studies in animal models are usually performed using single strains, or strains that are administered as supplements, and thus the effect of the food matrix itself is ignored. Finally, preclinical testing is generally omitted, and consequently, many new functional products containing single or multi-strain consortia are tested in clinical trials without prior knowledge of their mechanism of action. By using this approach, strains seem to be selected on a random basis to elicit favorable outcomes. Adding to that, other methodological issues in the measurement of outcomes, such as qualitative parameters or self-reported outcomes, could seriously undermine the analytical rigor of the studies [63]. Metanalyses could remedy this problem; however, usually a small number of available studies using the same experimental design are available, thus performing metanalyses can lead to several pitfalls. Some of these studies often use the word ‘probiotics’ as an umbrella term to lump together studies that investigate the effect of different supplements or functional foods against a specific outcome; consequently, the concept of species- and strain-specificity of actions is completely disregarded. Additionally, in the case of functional foods, seemingly identical products (e.g., fermented milk products) can differ drastically in their microbial and chemical composition, and thus comparison between studies that do not disclose these two components could be misleading.

As functional foods are generally regarded as safe for consumption, they can make their way into the clinic and human studies more easily. Therefore, important steps for the characterization of the properties of these foods and microorganisms are generally omitted. From this perspective, we would like to underline the importance of the characterization of strains and their potential biological actions on the host, at multiple levels, prior to clinical testing. The high accessibility of sequencing platforms and predictive biology algorithms can support this objective. Indeed, gene mining can inform us about the metabolic preferences and biosynthetic capability of strains, about microbe–microbe interactions and quorum sensing, or about the production of antimicrobial compounds that could burden the food or gut microbiome [65,66,67]. In this context, trophic network analysis can also provide insights into mechanisms of co-dependency or antagonism between microbes [27]. Furthermore, transcriptomic, proteomic, and metabolomic analysis of the strains after introduction to the food microbiome or gut niche could give important insights about environmental pressures and aid in the prediction of their phenotypic characteristics in different microenvironments. As the integration of these data could be a tricky task, bioinformatic pipelines have evolved along with these tools to support the meaningful interpretation of results.

Multi-omic platforms offer a unique opportunity to characterize novel strains of interest and microbe–microbe and microbe–matrix interactions before their incorporation in foodstuffs. Importantly, the same tools and platforms can be applied when studying the host’s response to the consumption of functional foods. A handful of studies have already incorporated such approaches. More specifically, a clinical study investigated the effect of a probiotic yoghurt that was fermented by *Lactobacillus delbrueckii* spp. *bulgaricus*, *Streptococcus thermophilus* (Thermophilic Yoflex^®^ culture, CHR Hansen, Hørsholm, Denmark), and *Lacticaseibacillus rhamnosus* GG (LGG) on postprandial lipid metabolism in a crossover clinical study. RNA sequencing of the peripheral blood transcriptome revealed that the intervention resulted in modulation of gene sets involved in metabolism and inflammation [68]. Importantly, whole blood transcriptomic analyses highlighted mild changes in the global gene expression of the host and revealed significant changes in biomarkers that are not regularly included in clinical studies [68]. As such, the main advantage of using these platforms is that they can provide a global snapshot of the expression of human cells after microbial stimuli. The meaningful interpretation of the extracted data can result in the construction of networks to map probiotic effects on the host [46]. Furthermore, these global, unbiased approaches can reveal unexpected results and generate new leads for microbe–host interactions. The systematic characterization of these relations and the underlying mechanisms of probiotic action could pave the way to targeted, host-specific supplementation with maximized efficacy.

## 7. Conclusions

Functional products are popular among consumers due to their enhanced organoleptic characteristics and potential health benefits. However, despite the volume of available in vitro and in vivo data on their health-promoting properties, there is limited translatability of research findings in the clinical setting. Furthermore, the lack of consistency in existing clinical data to support the beneficial actions of these products highlights the need for further elucidation of their biological effects. In this vein, the characterization of bioactive compounds and metabolic byproducts present in the food matrix could illuminate their biological potential and support targeted supplementation. Accordingly, the study of population dynamics—the interplay between probiotic microorganisms, host, and food microbiome—is pivotal to reveal antagonistic relationships and trophic networks formed by strains, thus paving the way to more efficacious interventions. The incorporation of multi-omic platforms and the meaningful interpretation of clinical data obtained from high-quality studies and proper outcome measures may contribute to the actualization of this objective.
